# A Meta-Analysis of the Efficacy of Case Management for Substance Use Disorders: A Recovery Perspective

**DOI:** 10.3389/fpsyt.2019.00186

**Published:** 2019-04-16

**Authors:** Wouter Vanderplasschen, Richard C. Rapp, Jessica De Maeyer, Wim Van Den Noortgate

**Affiliations:** ^1^Department of Special Needs Education, Ghent University, Ghent, Belgium; ^2^Boonshoft School of Medicine, Wright State University, Dayton, OH, United States; ^3^Centre of Expertise on Quality of Life, University College Ghent, Ghent, Belgium; ^4^Katholieke Universiteit Leuven Kulak, Kortrijk, Belgium

**Keywords:** case management, addiction, systematic review, effectiveness, treatment

## Abstract

**Background:** Case management is a client-centered approach to improve the coordination and continuity of service delivery, especially for persons with substance use disorders (SUD) and multiple and complex support needs. This intervention supports individuals by helping them identify needed services, facilitate linkage with services, and promote participation and retention in services. However, it is questionable whether case management is equally effective in promoting recovery and aspects of personal functioning. The objective was to conduct an updated meta-analysis and to assess whether case management was more effective than treatment as usual (TAU) among persons with SUD for improving treatment-related (e.g., successful linkage with and retention in treatment) as well as personal functioning outcomes (e.g., substance use).

**Methods:** This meta-analysis focuses on randomized controlled trials (RCTs) that included persons with alcohol or drug use disorders and compared case management with TAU. To be eligible, interventions had to meet core case management functions as defined in the literature. We conducted searches of the following databases to May 2017: the Cochrane Drugs and Alcohol Specialized Register, CENTRAL, PubMed, Embase, CINAHL, and Web of Science. Also, reference lists of retrieved publications were scanned for relevant (un)published studies.

**Results:** The overall effect size for case management compared to TAU across all outcome categories and moments was small and positive (SMD = 0.18, 95% CI 0.07–0.28), but statistically significant. Effects were considerably larger for treatment tasks (SMD = 0.33, 95% CI 0.18–0.48) than for personal functioning outcomes (SMD = 0.06, 95% CI −0.02 to 0.15). The largest effect sizes were found for retention in substance abuse treatment and linkage with substance abuse services. Moderator effects of case management models and conditions were assessed, but no significant differences were observed.

**Conclusions:** The primary results from earlier meta-analyses were supported: case management is more effective than TAU conditions for improving outcomes, but this effect is significantly larger for treatment-related tasks than for personal functioning outcomes. Case management can be an important supplement to available services for improving linkage and retention, although further research is needed to assess its potential for supporting recovery from a longitudinal perspective.

## Introduction

### Rationale

Substance use disorders (SUD) are associated with a wide range of consequences, including adverse health, social and economic outcomes (1–4). The health status of persons with alcohol and drug problems is often negatively affected by their substance abuse and SUDs contribute significantly to the global burden of disease (4, 5). Consequently, life expectancy and disability adjusted life years are often much lower among this population (6, 7). The co-existence of SUDs and other psychiatric disorders is widely documented and poses specific treatment challenges (8). Moreover, people with alcohol or drug use problems are more likely to be negatively affected on key employment measures such as being employed (9), maintaining productivity, and remaining in the workforce (10). Housing, judicial and relational problems are also pervasive among persons with SUDs, including a negative impact on partners, parents and children (11).

Persons with SUDs frequently have significant problems functioning in multiple areas of their lives, which seriously affects their social reintegration and recovery process (12). Some of these problems may have preceded substance abuse, or are direct results of it. In either instance, few treatment programs are equipped to provide the broad range of services necessary to meet the diverse support needs of this population (2, 13–15). SUDs are commonly recognized as chronic and relapsing disorders, requiring continuous support to promote recovery (2, 16, 17). The observation that many persons with SUDs have other lasting problems in addition to using substances was the main impetus for implementing case management as an addition to traditional treatment services from the 1980's onwards (18).

Following deinstitutionalization and the emerging recovery movement, case management was successfully adapted to the treatment and community-based support of various mental health populations in the United States, Canada, Australia and Europe (19–23). Its potential effectiveness for persons with SUDs was suggested in various narrative reviews (14, 18). Multiple randomized clinical trials of substance abuse case management have examined the intervention's impact on varied substance abusing populations: dually diagnosed persons, HIV infected drug users, opiate dependent individuals, female substance abusers, crack cocaine users, and homeless persons. Substance abuse case management has been adapted to work with persons in and out of treatment and in settings as diverse as treatment programs, emergency wards, welfare offices, correction and probation facilities, homeless shelters, and centralized intake units.

Case management is an intervention designed to enhance coordination and continuity of care and support, especially for persons with multiple, and complex needs (2). One of the first definitions described case management as “that part of substance abuse treatment that provides ongoing supportive care to clients and facilitates linking with appropriate helping resources in the community” [(24), p. 182]. Case management is an intervention that supports individuals by helping them “identify needed services, select the most appropriate services available, facilitate linkage with services and promote continued retention in services by monitoring participation, coordinating activities of multiple services when present and when necessary, and advocating for continued participation” [(25), p. 615]. In clinical trials, case management has been associated with over 450 different types of outcomes, which were clustered around 10 broad outcome categories in a meta-analysis focusing on studies published until 2011 (25). The association of case management with so many different outcomes suggests very unfocused expectations about where case management's value lies along the treatment continuum.

As in mental health care, several models of case management are identified, including brokerage, generalist, intensive, strengths-based, and clinical case management, as well as assertive community treatment (18). These different models facilitate the above-mentioned goals somewhat differently. *Brokerage case management* is intended to address some of these functions in a very minimalist manner in one or two contacts. Assessment, planning, linking, monitoring, and advocacy are core case management functions and central to *generalist* or standard case management. *Intensive case management* involves intensive contacts between case manager and client, although the extent of such involvement is not always specified. *Assertive community treatment* includes the provision of services by a multidisciplinary team, as well as referral to outside services and resources. *Strengths-based case management* focuses on utilizing individuals' strengths and assets and the use of informal rather than formal supportive networks. Finally, the *clinical model* of case management combines case management with clinical activities, for example psychotherapy and counseling (2).

### Objectives and Research Questions

Case management is likely to support the recovery process, but few studies have looked beyond substance use outcomes or included substantial follow-up periods. Also, findings from available systematic reviews are limited to narrative and global appreciations of study findings, repeatedly stressing its importance for improving individuals' overall functioning and—to a certain extent—substance use outcomes, and for enhancing linkage and retention (14, 18, 26–29). Meta-analyses offer additional opportunities to statistically synthesize data from various studies and to calculate effect sizes per study and outcome category, controlling for sample size, and various follow-up moments (30). Available meta-analyses of substance abuse case management are outdated (2) and/or focused on a variety of outcomes rather than effects of single studies (25).

This updated review will provide evidence either supporting or refuting the earlier findings, which will be discussed from a recovery perspective. The additional studies available for this review can also provide more details about moderators that might affect case management's efficacy. The objectives of this meta-analysis are threefold: (1) to assess the efficacy of case management for linking persons with SUDs with services they need and promoting treatment retention compared with ‘treatment as usual’ (TAU); (2) to evaluate whether case management positively impacts substance use and other life domains to a larger extent than standard treatment; (3) to study the role of potential moderating variables (e.g., type of population served, setting, model of case management, implementation fidelity affect case management outcomes). This review will address the critical questions of: (1) Is substance abuse case management efficacious compared with TAU; (2) Is case management equally effective in improving treatment task and personal functioning outcomes; (3) Is the effect of substance abuse case management the same across all outcome categories and models?

## Methods

### Study Design

This study is a meta-analysis of randomized controlled trials (RCTs) that have evaluated the efficacy of case management, reporting at least one follow-up measurement and one or multiple outcome indicators. Only RCTs that compared (a specific model of) case management with TAU were included. Studies were excluded if the randomization procedure was stopped or violated at some point, resulting in non-equivalent groups. In case the experimental and control condition received different pharmacological interventions, studies were excluded [see also (2, 29)].

### Participants, Intervention, and Comparators

The study sample consisted of persons with a SUD (abuse or dependence of any legal or illegal substance), not necessarily confirmed by a DSM diagnosis. Studies including subjects with other physical or mental health problems were eligible, if the entire sample had a SUD.

If manuscript authors called an intervention “case management,” the intervention was assessed based on the case management criteria developed by the US National Association of Social Workers (31). The proposed case management intervention had to meet at least four of the five functions of case management recognized by the NASW (assessment, planning, advocacy, linking, monitoring/evaluation) (25). In those instances where the intervention did not meet these criteria, it was excluded from the review. Interventions not labeled “case management” by manuscript authors could fit ≥4 NASW criteria and be included in the meta-analysis. Since case management has been applied for more than 30 years in the US (24), some trials have used it as part of a more comprehensive intervention for persons with SUDs (e.g., coordinated/integrated treatment) or combined with another intervention (e.g., motivational interviewing, vouchers or money to purchase treatment). Studies were excluded if it was impossible to disentangle case management effects from these of other interventions.

Only studies that compared case management with “treatment as usual” (TAU), as defined by the study authors, were selected. TAU may include various interventions called “standard of care,” “usual care,” or “standard treatment,” but generally refers to treatment as it is commonly provided. Case management has also been applied as a control condition in some studies, assuming its outcomes to be inferior than these of the experimental condition. Although inclusion of such studies could counter potential publication bias (32), these were excluded given the wide variation in type and intensity of case management as control condition and the loose description of these practices. Also, studies in which one case management model was compared with another were not included, since this was regarded a comparison of different modalities/intensities of the same intervention. In the absence of a non-case managed control condition, it was unclear which case management model should be regarded as control condition. Finally, studies that compared case management with clearly defined active (therapeutic, behavioral, or motivational) interventions [e.g., contingency management, motivational interviewing (25)] were beyond the scope of this review.

### Systematic Review Protocol

As case management is implemented among various populations with diverse objectives (2), it has been associated with hundreds of different outcomes. According to a review by Rapp and colleagues (25), case management's effectiveness has been evaluated across at least ten outcome categories, including over 450 different outcome measures. For the purpose of this review, the same 10 categories will be assessed, including diverse measures of each outcome. The first five categories relate to personal functioning outcomes and refer to changes in the behavior of persons with SUDs that are often reported in the recovery literature: reductions in substance use, risk behavior and legal involvement; improved health status and social functioning. The second group of treatment-related outcomes reflects the processes of treatment that can conceivably be affected by case management: linkage and retention in both substance abuse treatment itself and in referral to supportive, ancillary services.

1. Substance use (e.g., self-reported alcohol and drug use, biological markers, problem severity as measured by a standardized instrument).
2. Physical and mental health status (e.g., number of days in a hospital for physical/psychological problems, problem severity, quality of life).
3. Legal status (e.g., number of days incarcerated, problem severity).
4. Social inclusion, covering employment functioning, social and family relationships, and living situation (e.g., income from work, homelessness, problem severity, extent of the social network).
5. Risk behavior, including drug and sexual risk behavior.
6. Linkage (self-reported or administratively verified) with substance abuse services, including detox, outpatient, or residential treatment or aftercare).
7. Linkage with ancillary services that are supportive of other needs of persons with SUDs, such as housing, employment, mental health, and medical services.
8. Retention (self-reported or administratively verified) in substance abuse services (e.g., number of days of contact/treatment).
9. Retention in ancillary services (e.g., number of days of contact).
10. Satisfaction with treatment [e.g., individuals' satisfaction, acceptance or attitude about the treatment experience (in substance abuse and ancillary services)].

In case several outcome measures were reported in a given category, a single effect size was computed for each area per study, by averaging the effect sizes for each category (25). The outcome categories were assessed at all available follow-up moments and an averaged effect size per study across all follow-up moments was calculated.

### Search Strategy

Both electronic and manual searches were undertaken to identify papers, journal articles, research reports and book chapters for this review. We built on the search strategy of a previously published (withdrawn) Cochrane review (2) and updated this search by identifying relevant studies that met the predefined inclusion criteria in following electronic databases (search period January 2006 to May 2017): Web of Science (Thomson Reuters), EMBASE (Ovid), MEDLINE (PubMed), the Cochrane Drugs and Alcohol Group Specialized register and the Cochrane Central Register of Controlled Trials. We combined search terms that could identify the intervention (“case management”; “casemanagement”; “case managed”), population (“substance use disorders,” “addiction,” “substance abuse” or “dependence”), type of study (“randomized controlled trial”) and its focus (“efficacy,” “effectiveness,” “outcomes,” “evaluation”). Databases of ongoing clinical trials were also searched (www.isrctn.com and www.clinicaltrials.gov). We scanned the reference lists of retrieved reviews, journal articles, conference abstracts, and gray literature for other relevant (un)published studies (2). There were no language or publication year restrictions.

### Data Sources, Studies Sections, and Data-Extraction

Two authors (RCR, WVDP) independently screened the abstracts of all publications that were obtained through the search strategy. In case of disagreement, the study was assessed by a third author (JDM) and discussed between the three assessors. Two authors (RCR, WVDP) independently assessed the full texts of potentially relevant studies for inclusion. Again, any disagreement was resolved by involving a third author (JDM) and discussing eligibility between all three authors [see also (2)].

Two authors (RCR, WVDP) extracted data from the selected studies. WVDN checked all data extraction files. Any inconsistencies or obscurities were resolved by discussion between all three authors. Following information was extracted: number and characteristics of study participants, authors' names and country of origin, types of outcomes and potential conflicts of interest. Additional variables (see Table 1) were extracted that are particularly relevant for case management: model and location of case management, type of substance abusers, treatment status of participants upon study entry, presence/absence of a manual/protocol, or supervision, fidelity assessment (2). Also, the length of the follow-up period from which outcomes were presented was recorded.

**Table 1:** Characteristics of included controlled trials and studies.

Source + studies from same trial (indicated with^*^)	**Type of substance abuse**	**Case management location**	**Treatment status**	**Case management model**	**Comparison condition**	**Implementation fidelity**	
						**Supervision**	**Manual**
Martin and Scarpitti (36)	Polysubstance users	Criminal justice system (leaving prison on parole)	Out	Assertive Community Treatment (ACT)	Parole Only	No	Yes
Braucht et al. (37)	Polysubstance users (homeless)	Community	Out	Intensive CM	Residential Treatment Program	No	No
Zanis et al. (38)	Injectable drug users	Outpatient treatment (MMT)	In	Other (clinical)	Existing referral	No	Yes
Rhodes and Gross (7)	Polysubstance users	Criminal justice system (probation offices)	Out	Generalist CM	Existing referral	Yes	No
Cox et al. (39)	Alcohol dependent persons (homeless)	Community	Out	Intensive CM	Existing referral	No	No
Rapp et al. (40)	Polysubstance users	Outpatient treatment (entering aftercare)	In	Strengths-Based CM	Existing treatment (aftercare)	Yes	Yes
Siegal et al. (41)^*^							
Vaughan-Sarrazin et al. (42) In,Out	Polysubstance users	Residential and outpatient Treatment	In	Strengths-Based CM	Existing treatment	No	No
Saleh et al. (43) In,Out^*^							
Saleh et al. (44) In,Out^*^							
Vaughan-Sarrazin and Hall (45) In,Out^*^							
Saleh et al (46) In,Out^*^							
Hall et al. (47) In,Out^*^							
Scott et al. (48)	Polysubstance users	Community (Central Intake Unit)	Out	Generalist CM	Existing referral	Yes	No
Sorensen et al. (49)	Injectable drug users (HIV positive)	Community (hospital)	Out	Other (hybrid brokerage and full service)	Existing referral	Yes	Yes
Sorensen et al. (50)	Injectable drug users	Community	Out	Generalist CM	Existing referral	No	Yes
Barnett et al. (51)^*^							
Coviello et al. (52)	Injectable drug users	Community	Out	Generalist CM	Existing outreach	Yes	Yes
Jansson, (53)	Female polysubstance users (parents)	Community (hospital)	Out	Intensive CM	Existing referral	Yes	No
Morse et al. (54)	Polysubstance users (homeless + dually diagnosed)	Community (mental health centers)	Out	Assertive Community Treatment (ACT)	Existing referral	Yes	Yes
Morgenstern et al. (55)	Female polysubstance users	Community (welfare offices)	Out	Intensive CM	Existing referral	Yes	Yes
Morgenstern et al. (56)^*^							
Rapp et al. (57)	Male polysubstance users	Community (Central Intake Unit)	Out	Strengths-Based CM	Existing referral	Yes	Yes
Carr et al. (58)^*^							
Morgenstern et al., (12)	Polysubstance users	Community (welfare offices)	Out	Other (coordinated care management)	Existing referral	Yes	Yes
Morgenstern et al., (59)^*^							
Guydish, (60)	Female polysubstance users	Criminal Justice system (probation offices)	Out	Other (probation case management)	Probation only	Yes	Yes
Prendergast et al. (61)	Polysubstance users	Criminal Justice system (leaving prison on parole)	Out	Strengths-Based CM	Parole Only	Yes	Yes
Wu et al. (62)	Injectable drug users	Community	Out	Strengths-Based CM	Existing referral	No	No
Braback et al. (63)	Injectable drug users	Community	Out	Strengths-Based CM	Existing treatment	Yes	Yes
Drummond et al. (64)	Alcohol dependent persons	Community	Out	Assertive Community Treatment (ACT)	Existing referral	No	No

* *Indicates: study that is part of the afore-mentioned trial*.

As this is an update of a previously published review [(2), p. 5], we used the same protocol for data-extraction and extracted any relevant data for each of the outcome categories described above. For example, concerning drug use, if a study reported the ASI drugs severity and the number of abstinent days for each subject, we registered all data that allowed us to compute (averaged) effect sizes for each indicator. Data had to include either means or standard deviations for both the control and experimental group, a proportion for both the control and experimental group or statistics that allowed us to calculate an effect size, such as a univariate *F*-statistic, *t*-statistic, or a χ^2^-statistic with one degree of freedom. For each outcome measure, we recorded data on the degree of change in the experimental and comparison group, when available (2).

### Risk of Bias Assessment

Three authors (WVDP, RCR, WVDN) independently assessed the risk of bias in included studies. To make these judgements, the criteria indicated by the Cochrane Handbook for Systematic Reviews of Interventions were used (32). The domains of sequence generation and allocation concealment (avoidance of selection bias) were assessed by a single entry for each study. Blinding of participants, personnel, and outcome assessor (avoidance of performance bias and detection bias) was considered separately for objective (e.g., drop-out, substance use measured by urine analysis) and subjective outcomes (e.g., severity of withdrawal symptoms, self-reported use of substances). We included incomplete outcome data (avoidance of attrition bias) for all outcomes, except for drop-out from treatment, which is very often the primary outcome measure in substance abuse trials.

When several indicators reflecting the same construct are measured but only statistically significant effects are reported, publication bias may arise (2), leading to inflated overall effect estimates in a meta-analysis. The effect of publication bias (and therefore the inflation of the overall effect size) is likely to be larger for small studies: whereas for large studies even small observed effect sizes will be statistically significant, for small studies only the largest observed effect sizes will be statistically significant. We used visual inspection of funnel plots (plots of the effect estimate from each study against the sample size or effect standard error) to explore whether there is evidence for a negative association between the observed effect size and sample size. We want to note however that such a negative association is to be considered merely as an indication for publication or reporting bias, because it may be induced by other factors, such as an association between the study size and characteristics of the population or the intervention, or merely the role of chance. We inspected funnel plot symmetry when there were at least 10 studies included in the meta-analysis for a specific outcome measure.

### Data-Analysis

For each clinical trial, we calculated effect sizes separately for each outcome measure, as in the original meta-analysis [(2), p.5]. In case multiple indicators were reported that were relevant for a single outcome measure (e.g., number of drinks/day, days abstinent), we computed an effect size for each indicator separately, before averaging effect sizes per outcome. We also calculated all effect sizes separately for various follow-up moments. If feasible, measures with unknown or unsatisfactory psychometric properties were dropped from these analyses. Exceptions were: data from registers (e.g., treatment records, prison records) and objective data related to persons' living situation (e.g., employment status, receiving welfare benefits). Also, we used data from urine tests and other biological tests for analyses, even if no specific data on the validity of these tests were provided (2).

There was much variation in the way the results were reported in the primary studies. For continuous data, we used Hedges' g and corresponding standard errors, corrected for small-sample bias. If this information was not available from the primary studies, we made use of summary statistics (like means, standard deviations and group sizes) to calculate Hedges' g and standard error, or converted reported effect sizes (e.g., Pearson's r) or test statistics (e.g., *t*-statistics) to Hedge's g. For dichotomous outcomes, we used the log odds ratio (LOR) and standard error. If these were not reported, we calculated the LOR and standard error using information on odds ratios, cell frequencies, proportions and/or test statistics. Afterwards, these LORs were converted to Hedges' g. All calculations and conversions were done in Comprehensive Meta-Analysis (CMA) (33).

For the majority of the included trials, we had multiple observed effect sizes. One of the reasons is that for some clinical trials, we found effect sizes in multiple publications. This induced dependencies: effect sizes from the same study are likely to be related. We dealt with these dependencies by calculating the mean effect size for each study, before combining these averages in a traditional meta-analysis.

Heterogeneity was assessed by performing Q-tests for homogeneity. The between-study variance, tau^2^, was estimated as well. Moderator analyses were conducted to explore reasons for heterogeneity: when sufficient effect sizes were available (i.e., at least two per category), effect sizes were divided in categories and the effect of these categorical moderators was evaluated using the Q-test. By doings so, the effect of following categorical moderators was studied: case management model, treatment status (in/out of treatment), recruitment setting (community, welfare service, substance abuse treatment, criminal justice setting), drug use preference, and implementation quality (use of a manual/protocol and/or supervision).

Because the individual trials differ from each other in many aspects that may have an influence on the size of the effects, it is unlikely that the population effect sizes are exactly the same in all studies, not even after correcting for the influence of the moderator variables that were coded in our meta-analyses. Therefore, we made use of random effects models that take into account a possible (residual) heterogeneity in population effect sizes. To account for the risk of bias, we explored the moderating effects of allocation concealment (selection bias), blinding of outcome assessor (detection bias) and attrition bias. We performed sensitivity analyses by excluding trials with high risk of bias from the analyses. No differences were found for the primary outcomes between trials with a different level of risk of bias; when we excluded trials at high risk of bias for the three domains, it did not change conclusions.

## Results

### Study Selection and Characteristics

**Figure 1:**
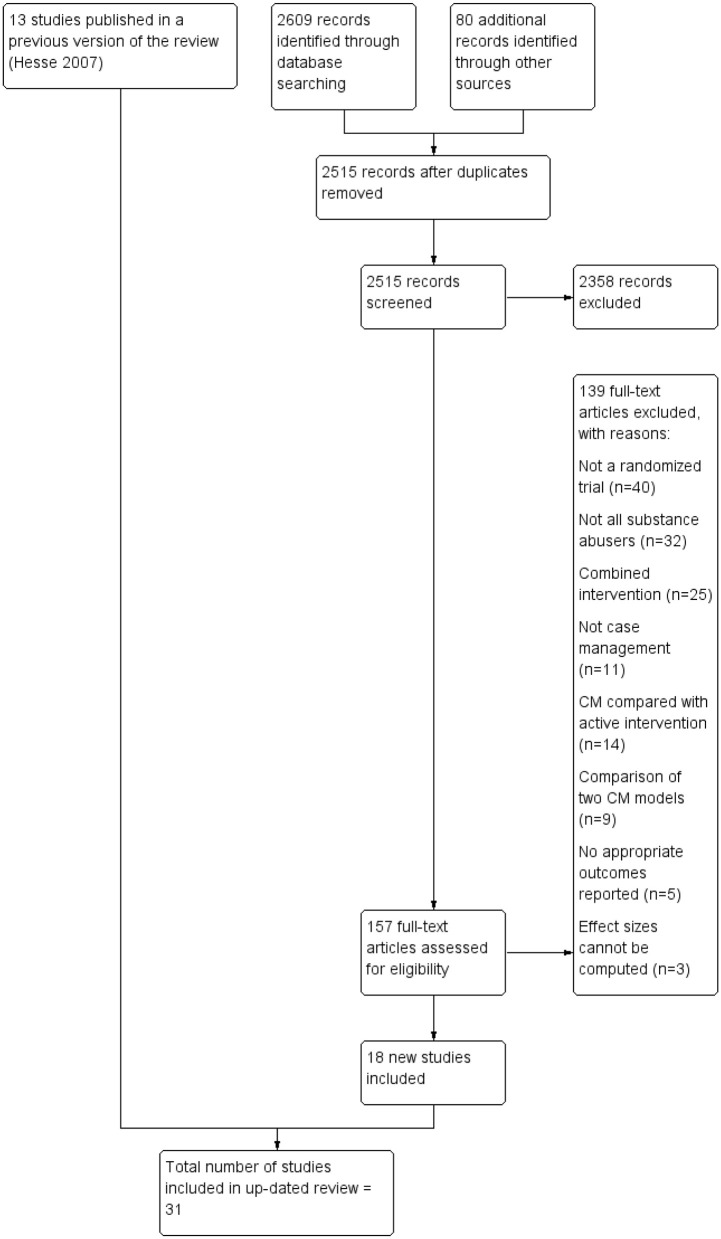
PRISMA study flow diagram.

Based on the search strategy outlined above, 2,515 unique documents were identified (see Figure 1). One hundred and fifty-seven (157) studies received in-depth screening, which led to the elimination of 139 studies for a variety of reasons. Reasons for exclusion were the following: the study was not a randomized clinical trial or not truly randomized (*n* = 40); not all of the study participants were persons with SUDs or at least some of the participants were children/adolescents (*n* = 32); case management was combined with another intervention and the effects of case management could not be separated out (*n* = 25), or the intervention did not meet the requirements for case management established in the protocol (*n* = 11). In other studies, two models of case management were compared (*n* = 9) or case management was compared with an active intervention rather than TAU (*n* = 14). In a few studies, reported outcomes did not conform to the study protocol (*n* = 5) and three studies that were otherwise eligible were excluded, because findings were not presented in a form that allowed calculation of an effect size.

The 18 eligible studies were combined with 13 studies from a previously published Cochrane review, resulting in 31 studies available for this meta-analysis. Two studies from the Cochrane review (*n* = 15) were removed as one appeared not randomized (34), while the other didn't compare case management to TAU (35). The 31 included studies were conducted as part of 21 different RCTs; some clinical trials generated more than one distinct published study.

Core characteristics of included trials and studies are outlined in Table 1 and are briefly described below. Sixteen of the 21 clinical trials resulted in one study/publication, four trials were published as two publications (40, 41, 51, 56–59, 65) and (55, 56) and one trial was reported in six studies/publications (42–47). Case management was most frequently compared with existing referral procedures (11 trials). Other trials compared case management with existing treatment (4 trials), parole supervision (*n* = 2), standard probation (*n* = 2), routine outreach (*n* = 1), or passive referral (*n* = 1).

A total of 7,431 unduplicated participants were randomized in the 21 clinical trials. The mean size of the trials was 354 subjects, ranging from a small study with 41 subjects (38) to a large trial including 1,369 subjects (7). Two typologies of substance use problems could be discerned: heroin or cocaine users involved in injectable drug use (IDU) (6 trials) and polysubstance abusers, that is, a mixed group of substance abusers where no one type of drug predominated (10 trials). Two trials contained a relatively homogenous substance abusing population consisting of individuals who were alcohol dependent (39, 64). Typically, study authors reported findings on specific subpopulations, including individuals who were homeless, dually diagnosed, HIV positive or female only (with children). Some of the study populations consisted of substance abusers involved in the criminal justice system: individuals on probation (7, 60) or persons on parole (36, 61). The Morgenstern trials (12, 55) involved substance users in public welfare settings, while two trials recruited individuals in central intake units (48, 57). At the time they entered the study, the majority of study participants were not in treatment. Only three clinical trials were composed exclusively of in-treatment substance abusers (38, 40, 42) (see Table 1).

Several case management models have been identified in the literature [see (18)]. The term “generalist case management” was assigned to four clinical trials that did not specify a conceptual or working name for the model of case management that was used. The term “intensive case management” was retained for the four trials using the term, although none specified what the term “intensive” meant. Six trials described case management as “strengths-based,” three as “Assertive Community Treatment” (ACT) and four were labeled as “other”, as they referred to case management using an unusual term: hybrid case management (49), outreach case management (38), coordinated care management (12, 59) or probation case management (60). Ten of the selected trials reported using both supervision and a manual/protocol to monitor quality control and fidelity of the case management intervention. Three trials used a manual, but not supervision, and conversely, three trials used supervision, but not a manual. Five trials used neither a manual nor supervision.

Out of the 7,431 unduplicated participants randomized in the 21 clinical trials, 6,179 were re-contacted at the first follow-up point, which means an overall follow-up rate of 83.2%. First follow-up moments for clinical trials ranged from 1.5 to 12 months for one of the studies. The modal first follow-up assessment was 6 months. Among the 21 clinical trials, seven had follow-up rates of 100% because information came from administrative records. Five trials had follow-up rates of 90.0–99.9%, 3 trials had rates between 80.0 and 89.9% and 5 trials had rates between 70.0 and 79.9%. One trial had unsatisfactory follow-up rates below 50%. Sample and follow-up rates were not used when follow-up data consisted of participants who had provided data at only one of multiple follow-up points. In some instances, it was not possible to identify exact sample sizes at each follow-up measurement due to unclear information provided in the retrieved publications.

### Synthesized Findings

**Figure 2:**
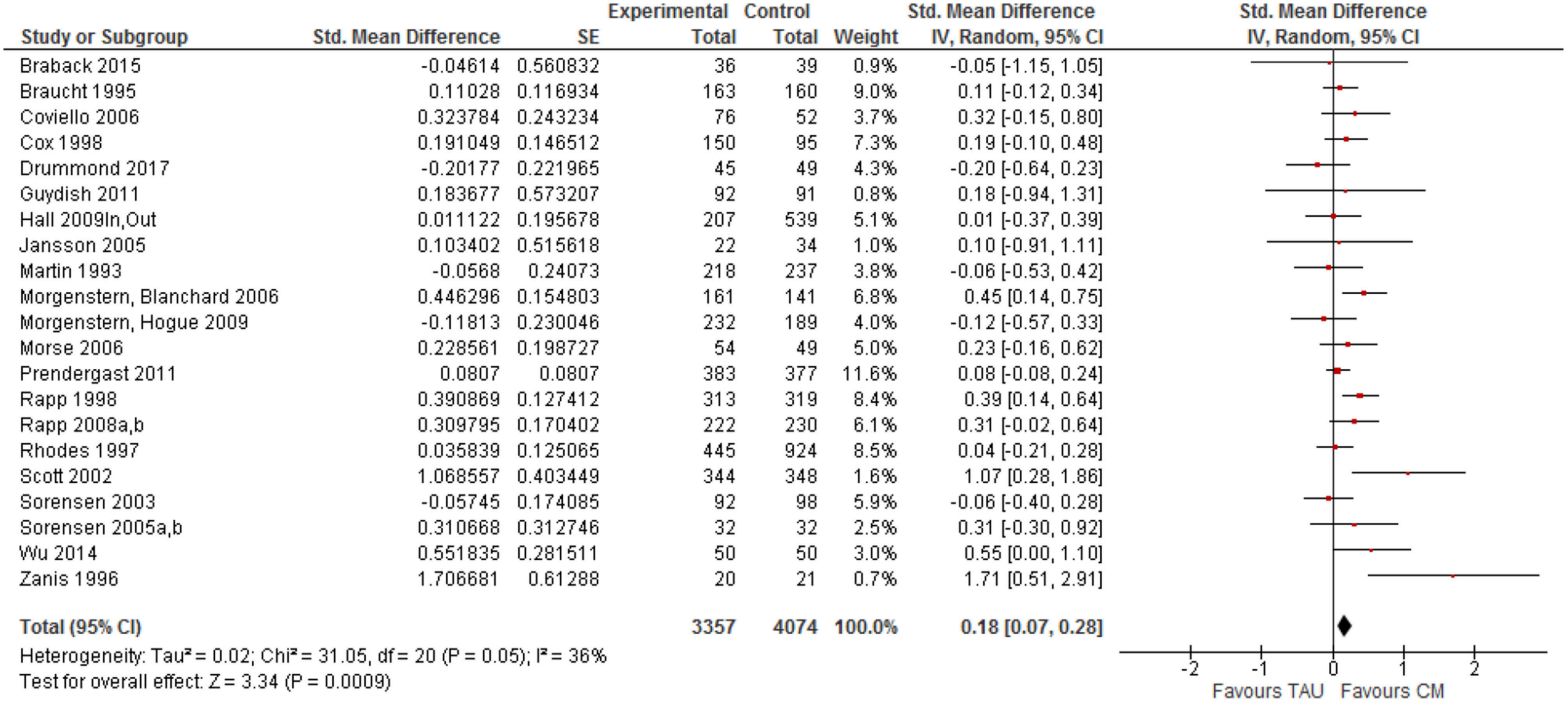
Forest plot of Case management vs. TAU: Overall CM effect across outcome categories.

All 21 clinical trials (31 studies in total) that reported a comparison of case management (CM) and TAU on one or more outcome indicators were included in the initial meta-analysis. The overall effect size for case management compared to TAU across all outcome categories and moments was small and positive, but statistically significant (*z* = 3.34, *p* < 0.001) with a mean effect of SMD = 0.179 (95% CI [0.07–0.28] (see Figure 2). Observed effect sizes were positive for 16 of the 21 studies, with five exceptions (12, 36, 49, 63, 64). Overall effect size estimates for the clinical trials ranged from −0.202 (64) to 1.707 (38).

**Figure 3:**
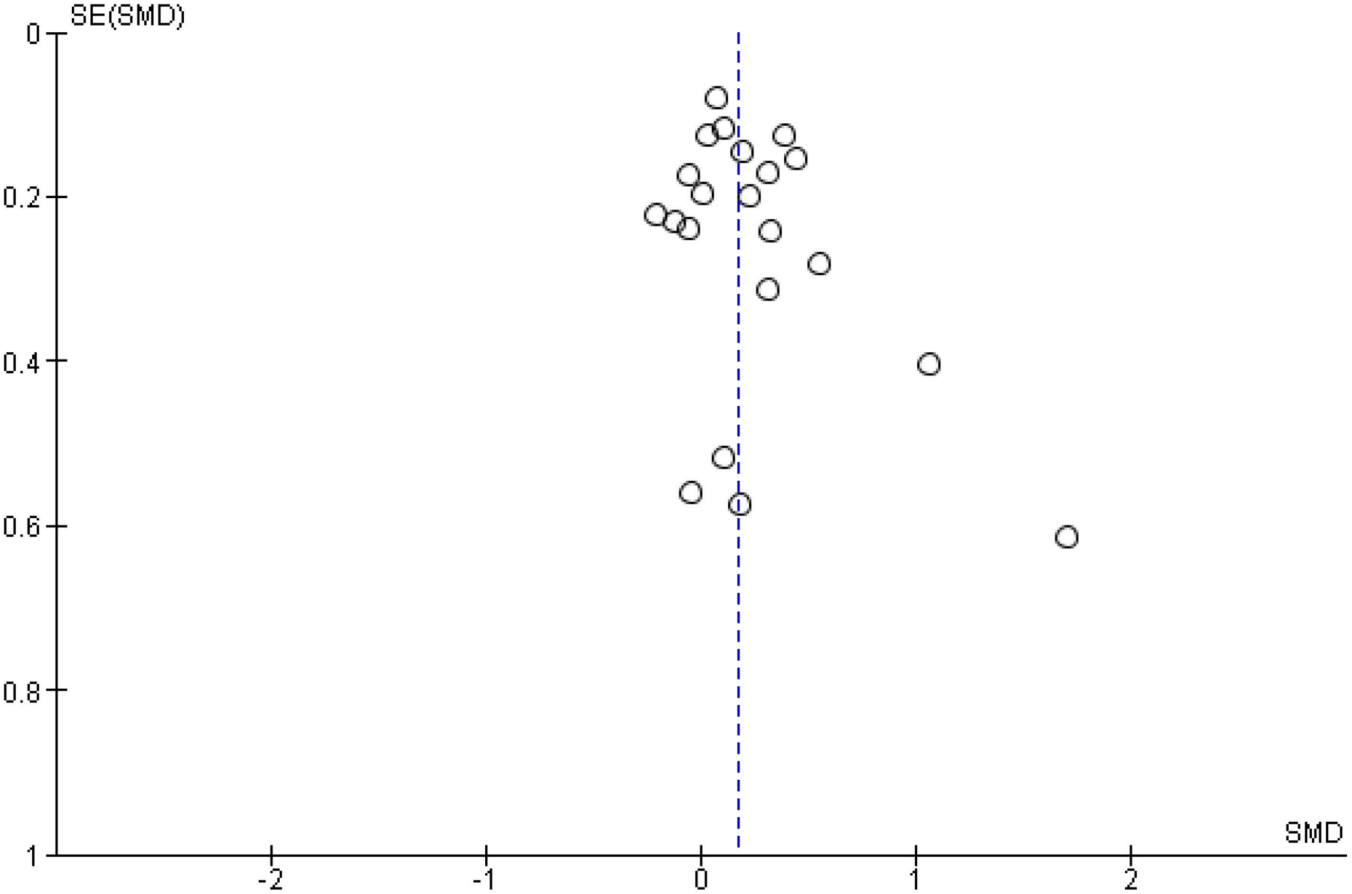
Funnel plot of Case management vs. TAU comparison: Overall CM effect across outcome categories.

There was little evidence for publication bias, as a visual examination of a funnel plot of standard errors appeared relatively symmetric (see Figure 3). Based on the trim-and-fill method, one effect size was added, and so the estimated overall treatment effect changed slightly (from 0.179 to 0.170). Egger's intercept test confirmed that there was little evidence for publication bias (*t* = 1.38, *df* = 19, *p* = 0.18).

We found some evidence for heterogeneity between studies. The estimate of the systematic between-study variance is equal to 0.018, *I*^2^ is equal to 35.59. This means that about 36% of the variance in observed effect sizes does not seem due to random sampling variance, but rather to systematic variation between studies. Despite this relatively large proportion of variance, the Q-test showed that this variance is statistically not significant when using a significance level of 0.05 (*Q* = 31.05, *df* = 20, *p* = 0.06). This does, however, not exclude the possibility that there are moderator variables affecting case management outcomes. Therefore, we performed several moderator analyses.

The effect of case management was studied using 10 different outcome types that were categorized into two broad groups: (1) treatment tasks (linkage with substance abuse and ancillary services, retention in substance abuse and ancillary services, and attitudes toward treatment) and (2) personal functioning outcomes (substance use, health status, legal involvement, risk behavior, and social functioning) (25). Of the 21 trials, 19 contained treatment task outcomes, and 15 trials reported personal functioning outcomes. Two separate meta-analyses were performed for these two categories of outcomes.

#### Case Management and Treatment Tasks

**Figure 4:**
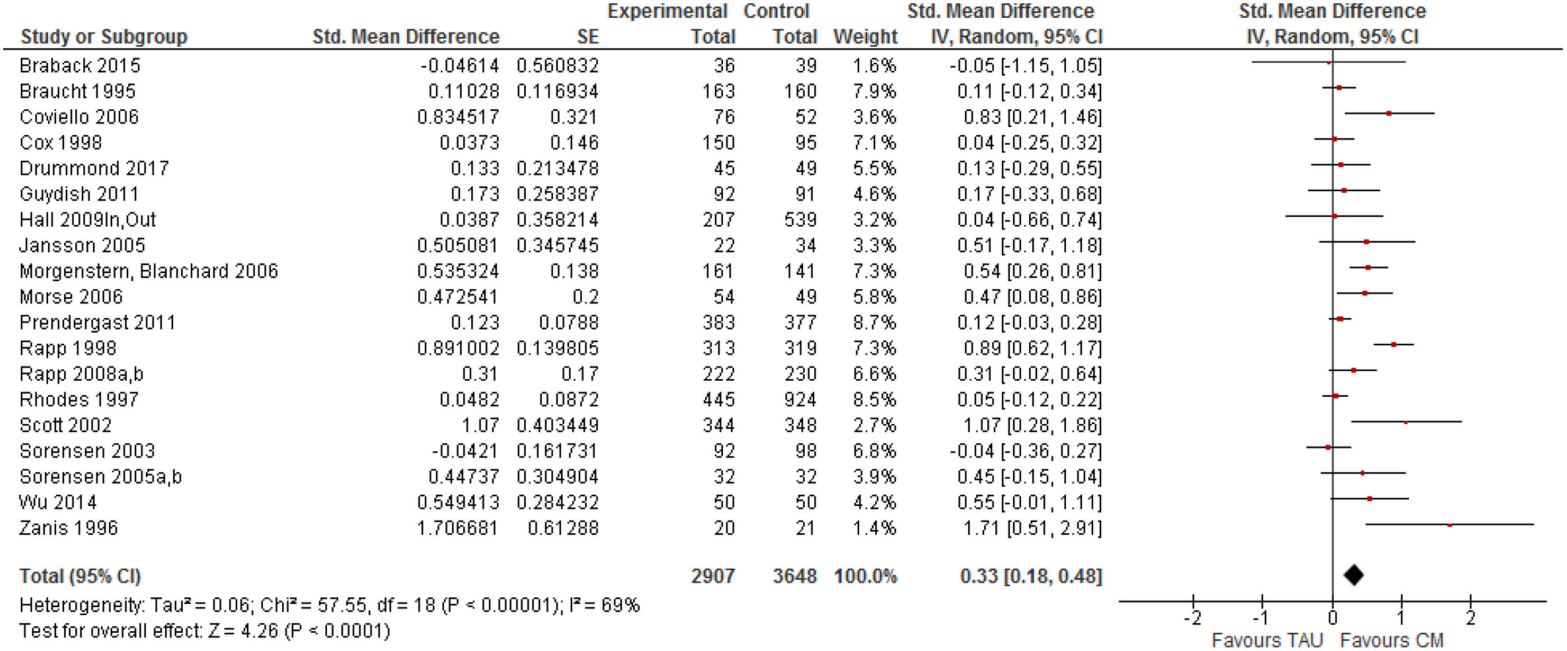
Forest plot Case management vs. TAU: Treatment related tasks.

For treatment tasks, a positive effect size was found in 17 (out of 19) trials ranging from 0.037 (39) to 1.707 (38) (see Figure 4). Only two trials showed a (very small) negative effect size for treatment tasks (49, 63). Seven of the trials had effect sizes above 0.5, what can be considered as a moderate effect (66). Overall, a weak to moderate effect of CM was found regarding treatment-related tasks, SMD = 0.33, 95% CI [0.18, 0.48]. Again, no clear evidence for publication bias was found.

Based on separate meta-analyses, we estimated the effect sizes for each of the five treatment tasks. The largest effect size was found for retention in substance abuse treatment (SMD = 0.47, 95% CI [0.13, 0.81]). Smaller effect sizes were found for linkage with substance abuse services (SMD = 0.23, 95% CI [0.11, 0.35]), satisfaction with treatment (SMD = 0.17, 95% CI [−0.04, 0.38]), retention in non-substance abuse services (SMD = 0.12, 95% CI [−0.01, 0.25]) and linkage with other types of services (SMD = 0.11, 95% CI [−0.11, 0.34]).

#### Case Management and Personal Functioning

**Figure 5:**
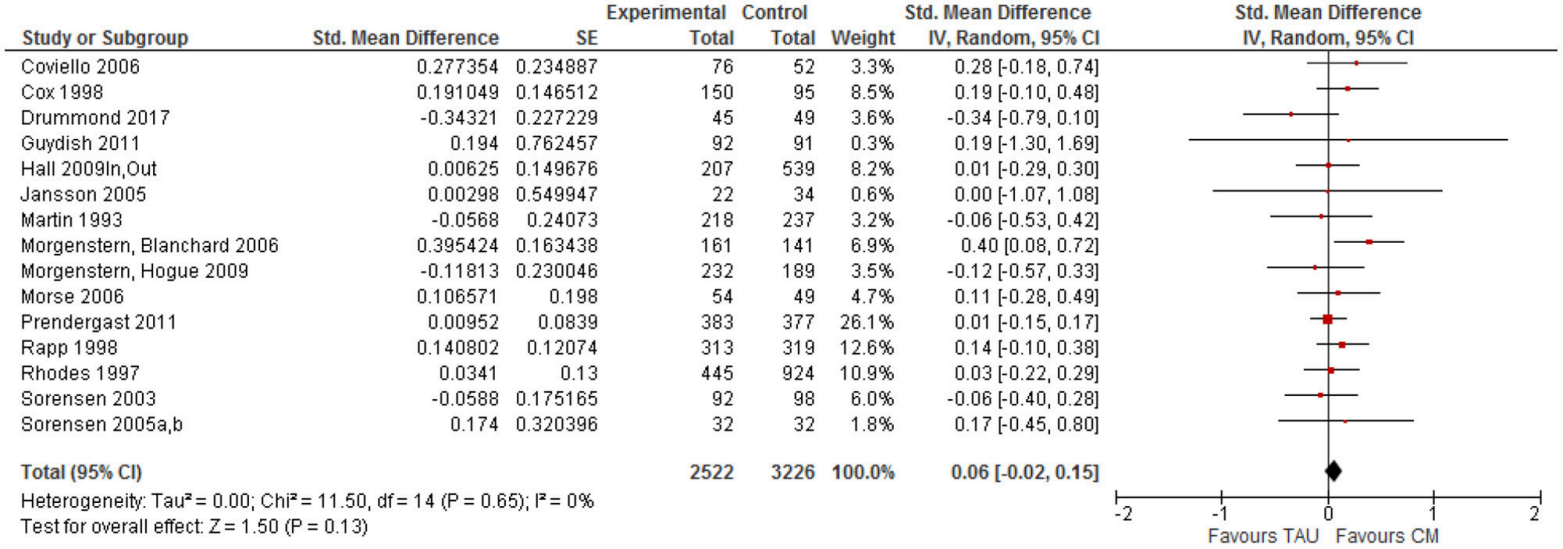
Forest plot of Case management vs. TAU: Personal functioning outcomes.

Of the 15 clinical trials with personal functioning outcomes, all but four had positive effect sizes that ranged from 0.003 (53) to 0.608 (62) (see Figure 5). Three studies had a very small negative effect size for functioning outcomes (12, 36, 49), but one study reported a negative effect size of −0.34 (64). Effect sizes were in general very small, including only three trials (39, 52, 55) with an effect size >0.20 (66) and two other studies (39, 60) with an effect size around 0.20. The overall SMD for personal functioning outcomes (SMD = 0.06, 95% CI [−0.02, 0.15]) was very small and statistically not significant. A funnel plot does not give evidence for publication bias.

Based on separate meta-analyses, we calculated the effect sizes for the five personal functioning outcomes. The biggest, although small and non-significant effects were found for social functioning (e.g., housing, employment) (SMD = 0.14, 95% CI [−0.01, 0.28]) and substance use outcomes (SMD = 0.10, 95% CI [−0.02, 0.21]). Even smaller (positive) effects favoring case management were found for risk behavior (SMD = 0.05, 95% CI [−0.07, 0.17]) and legal involvement (SMD = 0.02, 95% CI [−0.07, 0.10]), while a similarly small negative effect was found regarding health outcomes (SMD = −0.16, 95% CI [−0.40, 0.08]). None of these effects, however, were statistically different from zero.

#### Moderators

Six moderator variables were tested for their impact on the efficacy of case management, i.e., model, treatment status, setting, population, and intervention quality (manual or supervision).

**Figure 6:**
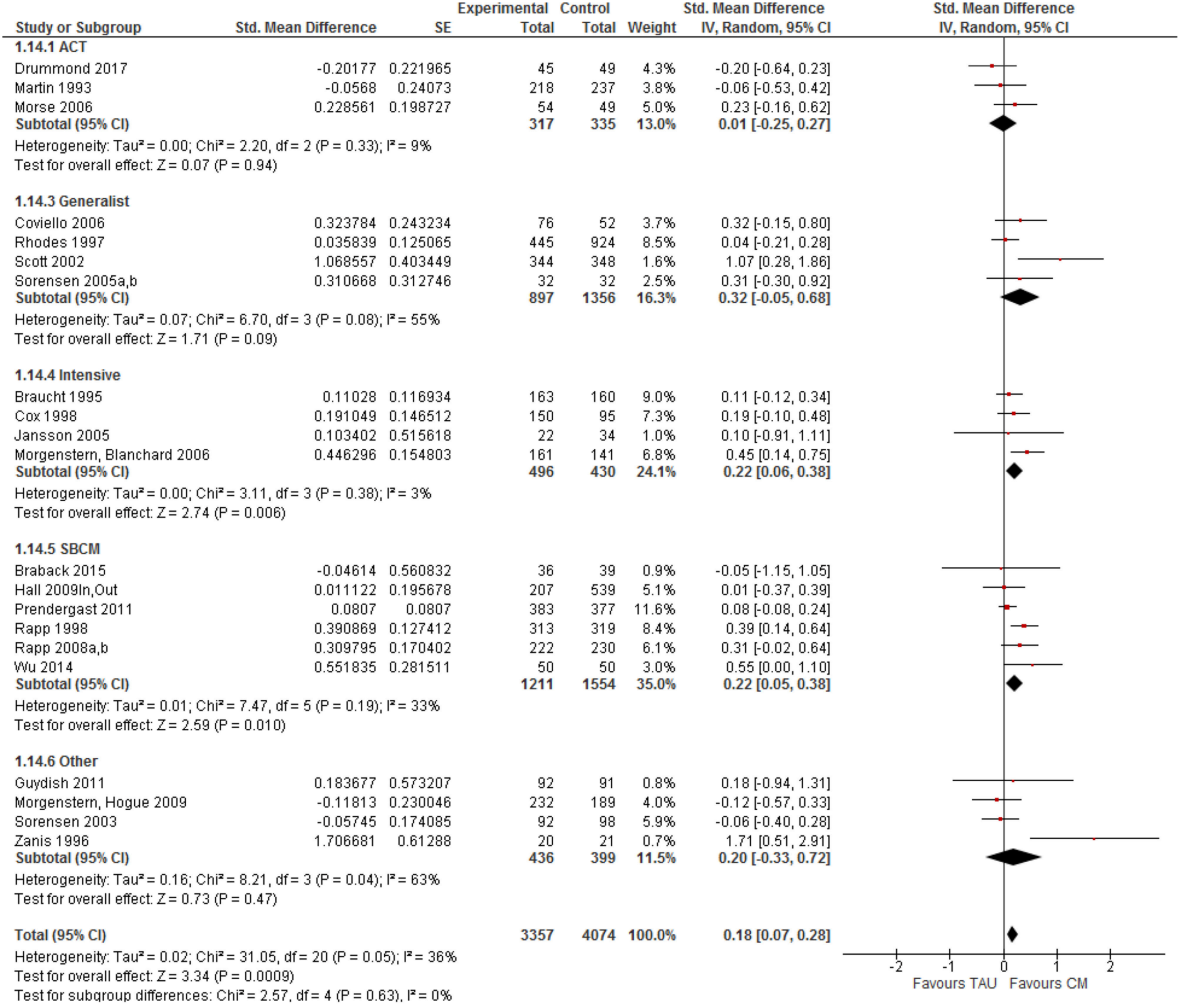
Forest plot of Case management vs. TAU according to CM model.

Studies were characterized by the type of case management model they used. The highest effect sizes were found for generalist CM (*k* = 4): SMD = 0.32, 95% CI [−0.05, 0.68], the lowest for Assertive Community Treatment (*k* = 3): SMD = 0.01, 95% CI [−0.25, 0.27] (see Figure 6). Estimated effect sizes were very similar for strengths-based CM (*k* = 6): SMD = 0.22, 95% CI [0.05, 0.38], intensive case management (*k* = 4): SMD = 0.22, 95%CI [0.06, 0.38], and for other types of CM (*k* = 4): SMD = 0.20, CI = [−0.33, 0.72]). Differences between CM models in the size of the intervention effect were statistically not significant (*Q* = 2.57, *df* = 4, *p* = 0.63).

Included studies were categorized as having participants who could be either “in” or “out” of treatment when the intervention started. Because the treatment status could vary within studies, we performed two separate meta-analyses. If the treatment status was ‘in’, the estimated mean effect was 0.18 (*k* = 3). If the treatment status was “out,” the overall effect size was very similar (SMD = 0.17, *k* = 19).

Studies were characterized as having participants in one of four types of settings at the time the study started: community/street (SMD = 0.16), criminal justice system (SMD = 0.20), substance abuse treatment (residential, outpatient, or mixed) (SMD = 0.23), or welfare offices (SMD = 0.19). Differences between these four categories appeared to be very small and statistically not significant (*Q* = 0.24, *df* = 3, *p* = 0.97).

Studies were characterized by the primary type of substance use, either alcohol, poly substance use or IDU. Although the estimated effect size for studies targeting injectable drug users was considerably higher (SMD = 0.33 for IDUs vs. 0.18 for poly substance users, and 0.03 for alcohol abusers), the moderating effect of the type of population on CM outcomes was statistically not significant (*Q* = 1.29, *df* = 2, *p* = 0.52).

Clinical trials were identified as having methods in place to promote the fidelity with which the intervention was implemented, one of these being a manual/protocol and the other clinical supervision. No significant difference in estimated effect size was found between studies with (SMD = 0.20) and without supervision (SMD = 0.16) (*Q* = 0.10, *df* = 1, *p* = 0.75). Also, no significant difference was found between studies with (SMD = 0.17) and without manual (SMD = 0.19) that described the intervention in detail. Effect sizes were very similar (*Q* = 0.05, *df* = 1, *p* = 0.83).

### Risk of Bias

We assessed all included studies (*n* = 31) for risk of bias, since several clinical trials included ≥1 publication/study that focused on specific outcomes or follow-up moments. We distinguished between following types of bias: selection bias, performance bias, attrition bias, detection bias and reporting bias [see (32)]. For each type of bias, studies were judged to be at “low,” “high” or “unclear” risk of bias.

Eighteen studies (11 clinical trials) reported an adequate randomization method and were judged to be at low risk of bias for “Random Sequence Generation,” 10 studies (4 clinical trials) were at high risk of bias, while the risk was unclear in 3 studies. Four early studies (7, 36, 37, 42) did not apply a systematic method of sequence generation. Similarly, 18 studies (13 clinical trials) described adequate concealment of the allocation sequence and were at low risk of bias for “Allocation Concealment,” 8 studies (4 clinical trials) did not report an adequate procedure for allocation concealment and in 5 studies this risk was deemed unclear. Due to the type of intervention, participants and staff could not be blinded for receiving/providing case management. Still, blinding of participants and personnel (performance bias) was assessed to be at low risk of bias in all studies (21 trials), as it was deemed unlikely that knowledge of receiving case management will have affected outcomes. Researchers reported blinding of outcome assessors in 20 studies (15 clinical trials), which were assessed to be at low risk of detection bias. The risk of detection bias was high in 6 studies (2 clinical trials), and in 5 studies the risk was unclear. Follow-up rates were high in most studies, because at least part of the outcome data were collected using administrative records. One trial had very low follow-up rates (<50%) and 15 trials had a high follow-up rate ≥80%. Attrition bias was judged as high risk in 11 studies (6 clinical trials), primarily due to follow-up rates <70% for at least some outcome areas. The risk of within-study selective outcome reporting was deemed low in most studies (*n* = 23; 17 trials), while high risk of reporting bias was observed in 7 studies (3 clinical trials) and “unclear” in 1 study.

## Discussion

### Summary of Findings

The current study examined the effects of case management regarding various indicators of recovery and service utilization, updating results from two previous meta-analyses (2, 25) and adding effect sizes for various types of outcomes to available systematic reviews (14, 27–29). Outcomes were clustered around 5 personal functioning and 5 treatment-related outcomes as described elsewhere (25). The primary results from the earlier studies were supported: case management was significantly more effective than TAU conditions for improving outcomes, although the overall effect was small (SMD = 0.18). The effect size was significantly larger for treatment related tasks (SMD = 0.33) than for personal functioning outcomes (SMD = 0.06), questioning its additional value in individuals' recovery process. However, substantial heterogeneity was observed between as well as within studies.

The findings suggest a positive role for case management over standards of care, but two factors make it difficult to fully understand the findings. First, numerous diverse outcome indicators (>450) are presented in clinical trials, including reduced substance use and criminal involvement, improved parenting skills and overall well-being, and linkage with treatment. Such broad expectations of a single intervention seem unwarranted, “as it is unlikely that any single psychosocial intervention can affect so many different areas of participants' lives” [(25), p. 614). Second, the TAU comparisons varied widely in their intensity, providing very different comparisons to case management. In ten of the trials, the TAU comparison was “existing referral practices,” a broad category that was usually ill-defined. In three trials, the comparison condition was either residential or aftercare treatment, both of which would quite possibly be more intensive than case management. Finally, in four trials TAU actually consisted of probation or parole, which is certainly an intensive comparison condition given the possibility of individuals being incarcerated. The finding that case management had a weak to small effect across all outcomes, even when some of the comparison conditions were relatively intense, suggests that the reported effect sizes are conservative and would have been larger if case management was compared to no treatment or waitlist controls (25).

### Differential Efficacy for Treatment Task and Personal Functioning Outcomes

Previous meta-analyses have suggested a differential effect of case management based on the type of outcomes being considered. One group of outcomes—treatment tasks—focused on the process of treatment, that is, do individuals link with and stay involved in both substance abuse treatment and ancillary substance abuse-related services. Attitudes toward attending treatment are also part of treatment task outcomes. The second group of outcomes consists of aspects of psychosocial and behavioral functioning that are generally the primary focus of substance abuse treatment. These areas encompass improved functioning regarding substance use, physical and psychological health, legal involvement, social status (housing, employment, family relations), and risk behavior. A clinically important and statistically significant difference was observed in case management's impact on treatment task outcomes compared to personal functioning outcomes [see also (2, 25)]. Effect sizes for the highest personal functioning areas, social functioning and substance use, were considerably lower compared with effect sizes for following treatment tasks: retention in substance abuse services and linkage with substance abuse and ancillary services.

Similar results were found in meta-analyses of case management with other mental health populations (21, 67, 68). Given the primary goals of case management (to help individuals identify needed services, facilitate linking with these services, monitor treatment participation and retention, coordinate service provision, and advocate on clients' behalf) (31, 69), its greater effect on treatment tasks is not surprising. The findings support an obvious premise: if individuals with SUDs do not link with and remain in treatment, especially in substance abuse services, they cannot benefit from these services (25). Consequently, case management should be regarded as a missing link in substance abuse treatment (24, 70).

### Case Management's Role in Supporting Recovery

The emerging international (mental health) recovery movement has stressed the importance of personal and subjective experiences of recovery [e.g., (71, 72)], besides “clinical recovery.” The latter refers to the absence of symptoms and illness ((i.e. most personal functioning outcomes) (73, 74), while “personal recovery” refers to a deeply personal process of change and “living a satisfying, hopeful and contributing life, within the limitations imposed by illness” [(75), p. 15]. Addiction recovery has been defined in more behavioral terms and is characterized as involving control over substance use, global health and well-being, and active citizenship (or community participation) (76, 77). Only a few studies selected for this review have included measures of personal recovery like quality of life and overall daily functioning, despite the recognition of SUD as a chronic relapsing disorder (17). The effect of case management on personal functioning outcomes found in this meta-analysis was weak to small, and only for employment and housing outcomes (social functioning, SMD = 0.14) and substance use (SMD = 0.10) a weak effect was found. A weak, negative effect was found for health outcomes (SMD = −0.16), a combination of physical and mental health indicators, typically measured from a clinical recovery perspective. Recovery should be regarded as a long process involving several life domains and abstinence is not a necessary, nor a sufficient marker of recovery, as it concerns a personal and experiential journey to life satisfaction and well-being (72). The transition from early stages of recovery to ‘stable recovery’ averages around 5 years, with the recognition that there are multiple pathways to recovery (78).

All models of case management have in common the goal of linking persons with SUDs and their families with needed resources in order to promote personal functioning and recovery (57). Linking with and effective use of community resources and services addresses their needs for substance abuse treatment, safe housing, improved employment, management of health problems, and avoiding legal problems. Resolution of these problems should increase individuals' opportunities to effect recovery. Case management further promotes that persons with SUDs keep on using these services and stay in treatment (14, 28, 41). Consequently, case management is thought to directly affect treatment-related outcomes (e.g., linkage, retention) and, by doing so, to indirectly impact on personal functioning outcomes (e.g., alcohol and drug use, employment, health status, family relations) and thus recovery.

Although case management was not found to be directly associated with improved personal functioning, two other mechanisms may be in operation (14, 25, 56). First, case management may have an indirect effect on separate personal functioning outcomes and overall recovery through its impact on treatment tasks such as linking and retention. Treatment participation and retention are widely documented predictors of remission and recovery (73, 79). For example, recently released parolees with SUDs who were receiving case management, were retained in treatment significantly longer than persons not receiving this intervention (60). Longer retention was associated with reduced substance use, less criminal justice involvement and risk behavior, and improved housing and employment situations at follow-up. Consequently, improved functioning should be viewed as a result of case management's ability to improve linkage and retention rather than as a direct effect.

Second, personal functioning outcomes—and eventually recovery—may be enhanced by combining case management with specialized skills and activities (e.g., a strengths approach) (25). Case management may include a variety of direct interventions, ranging from providing information and advice and substance abuse counseling to being clients' primary therapist in clinical models of case management (18). For example, clinical case managers receive specialized training to combine therapeutic support and case management (80). In this instance, it may be warranted to expect that case management contributes to improved personal functioning outcomes such as reductions in psychiatric symptoms and improved well-being (25, 81). Case management can also be combined with other interventions such as risk reduction activities, motivational interviewing, and recovery management [see (51, 62)]. Expanded case management services have been frequently applied among substance users with additional mental health (82, 83) and HIV/AIDS problems (49, 84). It is a standard part of comprehensive case management models like intensive case management (53, 55) and Assertive Community Treatment (ACT) (54, 64). Importantly, various studies were excluded from this review, as they offered comprehensive interventions combining case management with other viable approaches (e.g., cognitive behavioral therapy, Housing First programs), in which case management effects could not be disentangled from these additional interventions. Such promising combinations are worthwhile reviewing, although separating specific case management effects will be challenging. These observations illustrate that the variety between case management conditions (from brief models to comprehensive, long-term approaches) may at least be as diverse as the variety observed in TAU conditions, which is likely to affect case management's efficacy.

### Toward a Recovery-Oriented Research Agenda

The increased interest in case management resulted in the addition of several new trials (especially from outside the US) in this updated meta-analysis and over 130 recent studies (non-randomized, quasi-experimental, and observational studies) were excluded, primarily because they didn't apply a randomized study design or did not focus exclusively on substance users. Case management is often used to address severely disadvantaged populations with multiple and complex needs. This does not only challenge the randomization process in real-life settings and leads to the adoption of less rigid study methodologies (85), but also reduces the likelihood of finding large effect sizes (14). Also, the finding that case management is effective for improving linkage has contributed to its incorporation in comprehensive treatment programs (86–88) and to its acceptance as a mainstream intervention. In some clinical trials (89–91), case management was even applied as a standard of care control condition, assuming that this intervention would be inferior to the experimental intervention. These studies were not included in this systematic review, but should be reviewed in a separate meta-analysis that compares case management to other active interventions. Given the comparison with other viable (or even evidence-based) interventions, it is likely that case management's efficacy will be lower than compared with TAU.

Despite the increasing popularity of quality of life and other indicators of subjective well-being for evaluating interventions among persons with chronic disorders (92), such outcomes have hardly been studied in clinical trials of case management. Although these studies have often included self-report measures of health and substance use outcomes, they mostly refer to high expectations and socially desirable changes (e.g., abstinence from alcohol and drugs, no arrests, employment) rather than to individuals' perceived well-being and quality of life. Yet, such measures might shed an alternative light on individuals' situations and personal functioning outcomes (93). As case management is primarily applied among severely affected substance using populations, a focus on subjective and person-centered outcomes is more likely to demonstrate the benefits of case management in individuals' daily lives. Introducing a recovery perspective in controlled studies of case management will also allow to measure its impact on individuals' satisfaction with life and participation in society, as well as factors directly or indirectly affecting it. A number of trials were recently set up or are still ongoing that could potentially fill some of these gaps (94, 95).

### Quality of the Evidence and Limitations of This Review

According to the GRADE criteria (96), the quality of the evidence regarding the reported outcome categories would be rated moderate to low, primarily due to multiple risks of bias and substantial heterogeneity, or a combination of both. About 1 in 4 studies did not describe the randomization and concealment method adequately. Outcomes assessors were only blinded for group allocation in 2 out of 3 studies and most studies did not use blinding of study participants and case managers. When outcomes are self-reported behaviors and when participants are not blinded to study conditions, overestimation of intervention effects is a potential risk (97). However, since administrative data or objective outcome indicators (e.g., biological markers) were used in at least half of the trials, this risk was minimized. Attrition rates were not acceptable in one trial (42). Follow-up rates sometimes differed within one trial, as several studies used administrative records and assessments to evaluate study outcomes. Attrition bias may limit the applicability of study results or the power to detect between-group differences (97). Despite low attrition in some studies, this may not be problematic if attrition rates are similar in the experimental and control condition (97). We observed few indications for differential attrition rates in the included studies. Finally, publication bias may hamper the conclusions from any systematic review. Visual inspection of funnel plots suggested no evidence for publication bias, and the use of the trim-and-fill method only changed effect sizes slightly. Still, 1 in 5 studies were at high risk of reporting bias, as only some outcome indicators were reported in retrieved publications. Since this meta-analysis is based on published study outcomes, we may not underestimate the likelihood of publication bias as journals tend to publish studies including significant findings. Yet, the fact that several of the included RCTs generated no (or even inverse) effects favoring the case management condition illustrates that reported outcomes are not limited to significant positive outcomes.

This meta-analytic review has some limitations, most of which are typical for meta-analyses (98). First, all included studies were randomized clinical trials (25). Quasi-experimental trials are another source of evidence for case management studies, but these were excluded as we focused on studies in which all subjects were randomly assigned to either case management or a comparison condition. Second, even though all studies in this review were randomized, substantial methodological differences were observed (25). For example, studies used a variety of instruments to measure personal functioning outcomes, and at least 15 different substance use measures were used. This resulted in numerous different outcomes per outcome category. Third, fairly distinct populations were included in the clinical trials, e.g., persons with dual diagnosis, female welfare recipients, homeless alcohol dependent men, HIV infected intravenous drug users and incarcerated drug offenders. Even within these groups, study participants had diverse treatment needs, which may affect case managers' activities (14) Moreover, individual-level characteristics determine the effects of an intervention. For instance, there is evidence that SUDs are associated with various individual characteristics such as gender, comorbid disorders and psychosocial functioning [e.g., (99)], and it is possible that the likelihood of success of case management is associated with such factors. Ideally, this meta-analysis should have been based on the raw data of all individuals included in the selected clinical trials, so that we could perform a multilevel analysis accounting for covariates at the within-study level, in addition to covariates at the between-study level. By modeling the interaction effect of these individual characteristics and the intervention, we could have evaluated the moderating effects of these characteristics. In this meta-analysis, however, we had to rely on effect sizes summarizing effects for whole samples of participants, since the raw data were not available. It would still be interesting to study moderating effects of individual characteristics aggregated at sample level, but unfortunately, individual participant characteristics were not systematically measured and reported in the selected studies, so that we were not able to perform such moderator analyses (with the exception of the type of population studied). Therefore, part of the between-study variance in treatment effects may be due to differences in the composition of the samples in terms of participant characteristics. Fourth, in the absence of fidelity measures, we could not determine case management dosage or quality, nor could we control for eventual differences through moderator analyses (25). Fifth, we could not quantify all of the intervention features that might affect outcomes, although we used several characteristics of case management as moderators to help explain its efficacy. Case management is often characterized as being contextual and reflective of the network of services in which it is implemented (23), suggesting that the availability of services influences its overall efficacy (25). Consequently, the quality of service provision is likely to be beyond the control of case managers, even when resources are available. Finally, all study outcomes are characterized by substantial heterogeneity between and within study outcomes, which can be explained by the large variation in study populations, settings and treatment status, but also by within study variation regarding population, case management dosage and multiple follow-up measurements. We tried to control for some of these influences through moderator analyses.

## Conclusion

Three extensive meta-analyses, including this one, have now confirmed that substance abuse case management is efficacious in improving important treatment-related outcomes such as linking with and staying engaged (retention) in substance abuse and ancillary services compared with standards of care. Substance abuse programs often experience challenges in delivering and coordinating ongoing support and in providing access to additional services for persons with SUDs and multiple and complex problems. Enhanced linking and retention in substance abuse and ancillary services have been associated with improved abstinence rates (100), less frequent hospital readmissions (101), and adequate functioning in the community (102). Linking with and engaging in treatment are therefore necessary prerequisites to persons with SUD having an opportunity to benefit from these services. Although most models of case management seem to be equally effective in promoting these outcomes, this point is still not clear given the shortage of (comparative) trials of models of case management. Substance abuse case management did not have a significant direct effect on personal functioning outcomes compared with standards of care. The positive, but limited impact of this intervention on substance use and other clinical recovery-related outcomes is supposed to be mediated by case managed individuals' use of substance abuse and ancillary services and participation in treatment. Consequently, case management should be an integral part of comprehensive, wrap-around interventions to promote linkage and treatment participation and retention, and indirectly, personal functioning outcomes and recovery.

Further areas of research are at least 3-fold. First, studies that continue to include personal functioning outcomes should use methodological strategies that allow to assess the relationship between treatment linkage and retention on the one hand, and improved functioning outcomes on the other hand. Second, additional research is needed regarding some outcome categories (e.g., retention in and satisfaction with treatment), as case management is most likely to favor this type of understudied outcomes. Some type of outcomes have hardly been assessed in case management studies (e.g., quality of life, subjective well-being), although such indicators of personal recovery may enhance our knowledge substantially. Also, intensity and dosage of case management have been poorly studied. Third, despite the evidence that case management works for some specific purposes and populations, it remains largely unknown how it works and for whom at various stages of the treatment continuum. Future reviews should consider including quasi-experimental studies, since such designs usually resemble real-life settings more closely and the eventual question whether something works is whether it works in practice rather than under strictly controlled study conditions.

## Author Contributions

WVDP together with RCR selected the studies for this review, checked extracted data, assessed risk of bias in the selected studies, wrote the results and discussion section, and supervised this publication. RCR together with WVDP selected the studies for this review, extracted data from the selected studies and managed in- and excluded studies, assessed risk of bias in the selected studies and wrote the introduction, and methods section. JDM contributed to the search and study selection process and helped with writing the background and discussion section. WVDN calculated all effect sizes based on the extracted data, ran all meta-analyses, assessed various types of bias and revised the methods, and results section.

### Conflict of Interest Statement

RCR was involved in two of the RCTs included in this review (40, 57). The remaining authors declare that the research was conducted in the absence of any commercial or financial relationships that could be construed as a potential conflict of interest.
